# LncRNA PCAT19 induced by SP1 and acted as oncogene in gastric cancer competitively binding to miR429 and upregulating DHX9

**DOI:** 10.7150/jca.61961

**Published:** 2022-01-01

**Authors:** Lei Xiao, Weijie Yuan, Changhao Huang, Qingqing Luo, Runsha Xiao, Zi-Hua Chen

**Affiliations:** 1 Department of Gastrointestinal, Xiangya Hospital, Central South University, Changsha 410008, China.; 2 Department of Oncology, Hunan Provincial People's Hospital, Changsha 410002, China.

**Keywords:** LncRNA, PCAT19, Gastric cancer, SP1, miR-429, DHX9

## Abstract

Increasing evidence suggests that long non-coding RNAs (lncRNAs) are crucial in cancer biological processes. To investigate if lncRNA contributes to gastric cancer (GC), we conducted a bioinformatics analysis in human microarray datasets, and the results showed that lncRNA prostate cancer-associated transcript 19 (PCAT19) was upregulated in GC. Quantitative reverse-transcriptase PCR and *in situ* hybridization assays also revealed that PCAT19 was upregulated in GC tissues. The PCAT19 expression in GC was significantly related to tumor size, lymph node metastasis, and pathological stage. Moreover, patients with higher PCAT19 expression levels were more likely to have a poor prognosis for overall survival. The knockdown of PCAT19 by siRNA significantly suppressed the proliferation and invasion of GC cells. The cell distribution of PCAT19 in GC cells was examined by fluorescence *in situ* hybridization assay, and the results showed that it was mainly located in the cytoplasm. Mechanistically, PCAT19 sponges miR-429 and promotes DHX9 expression. In addition, the transcription factor SP1 is involved in PCAT19 activation. Our results demonstrate that lncRNA PCAT19 is induced by SP1 and acts as an oncogene in GC that competitively binds to miR429 and upregulates DHX9.

## Introduction

Gastric cancer (GC) is a leading malignant cancer that causes millions of deaths worldwide every year 1. Although there have been great improvements in GC treatment and diagnosis, the 5-year overall survival (OS) rate of patients has not significantly improved 2. Most GC patients are diagnosed at an advanced stage because the symptoms in the early stages are not typical 3. Moreover, the lack of effective molecular markers for the diagnosis and early metastasis further hinder the GC treatment. In this context, the molecular mechanisms underlying GC invasion and metastasis are poorly understood 4. Therefore, it is essential to identify molecular biomarkers as therapeutic targets in GC patients.

Long non-coding RNAs (lncRNAs) have more than 200 nucleotides but lack a protein-coding ability 5. Previous studies have demonstrated that lncRNAs are crucial for biological processes in tumors 6, and that they are correlated with cancer proliferation and poor prognosis 7-9.

Despite the wide research on lncRNA associated with GC, we speculated that there were still a number of lncRNA unexplored in GC. Therefore, we analyzed two independent microarray datasets of GC from Gene Expression Omnibus (GEO) databases. After a comprehensive analysis using bioinformatic methods, we identified 19 aberrantly expressed lncRNAs in both datasets. We then selected the prostate cancer-associated transcript 19 (PCAT19) as an upregulated lncRNA in GC for further study.

PCAT19 is a novel lncRNA that activates the cell cycle in prostate cancer progression 10, and it promotes the proliferation of laryngocarcinoma and non-small cell lung cancer 11. However, the function of lncRNA PCAT19 in GC has not been reported. In this study, we detected the expression level of PCAT19 and analyzed its correlation with GC prognosis. We demonstrated that the PCAT19 knockdown by transfected siRNA significantly suppressed the proliferation and invasion of GC cells. In addition, PCAT19 serves as a competing endogenous RNA (ceRNA) of miR-429, and it upregulates the DHX9 expression. We also observed that PCAT19 can be activated by the transcription factor (TF) SP1, and we revealed the molecular mechanism of PCAT19 in promoting the GC progression. The results suggest PCAT19 is a potential biomarker for future GC diagnosis.

## Materials and methods

### Clinical samples

All clinical samples were obtained from the Gastrointestinal Department of the Xiangya Hospital, Central South University. We collected the samples within 30 min of resection, and the study was approved by the Ethics Committee of the Xiangya Hospital. Major inclusion criteria were: (1) patients with pathologically confirmed gastric cancer; (2) patients with a negative history of any other malignant tumors; (3) patients that had not received any anti-cancer treatment. Major exclusion criteria were: (1) Patients with neoadjuvant chemotherapy; (2) patients with a positive history of other malignant tumors; (3) Patients with severe diseases in other organs. All patients in this study all gave informed consent from our teammates.

### Bioinformatics analysis

GSE106815 and GSE109476 gene expression data were downloaded from the GEO dataset. The normalized probe-level intensity files were also downloaded. The Affy package of the R language software was used for data background correction and normalization processing. We used probe sequences in GENCODE Release 21 sequence databases to determine lncRNA re-annotates. The differentially expressed lncRNAs were screened considering the thresholds of FDR < 0.05, and absolute fold change ≥ 2.0. Aberrantly expressed lncRNAs were identified using Venn analysis.

### Cell lines

All cell lines in this study were obtained from the Cell Center of Xiangya School of Medicine. The cells were cultured in a Roswell Park Memorial Institute (RPMI) 1640 medium (Invitrogen, Carlsbad, USA) containing 10% fetal bovine serum (Invitrogen, Carlsbad, USA). The cells were then stored in a culture incubator at 37 °C and 5% CO2 for further research.

### Cell transfection

Small interfering RNA (siRNA) was used to downregulate the PCAT19 expression (RiboBio, Guangzhou, China). PCAT19 and SP1 pcDNA were synthesized into the vector pCDNA3.1.

Approximately 2 × 10^6^ cells were plated in 6-well plates before transfection. When the density of the cells was approximately 50%, siRNA mixed with Lipofectamine 2000 reagent (Invitrogen, Carlsbad, USA) was transfected into the cells according to the protocol of the manufacturer. All siRNA sequences are listed in **Table S1**.

### RNA isolation and qRT-PCR

Total RNA was isolated using a TRIzol reagent (Invitrogen, Carlsbad, USA). Quantitative reverse-transcriptase PCR (qRT-PCR) assay was used to detect expression levels following the instructions of the manufacturer (HieffTM qPCR SYBR Green Master Mix, Yeasen, Shanghai, China). Glyceraldehyde-3-phosphate dehydrogenase (GAPDH) was used as the internal control, and the primers are listed in **Table S1**.

### Cell Counting Kit (CCK)-8 assay

Approximately 2000 GC cells were plated in 96-well plates, and cell proliferation was assessed after culturing for 24, 48, 72, and 96 h. Subsequently, 10 µL of CCK-8 reagent (Yeasen, Shanghai, China) was added to the cells, which were incubated for 2 h. Absorbance was measured at 450 nm using a microplate reader.

### 5-Ethynyl-20-deoxyuridine assay (EdU) Assay

Approximately 8000 GC cells were plated in 96-well plates. The cells were incubated with 50 mM of EdU for 2 h, following the protocol of the manufacturer (RiboBio, Guangzhou, China). The nuclei of the cells were stained with Hoechst for 30 min, and the percentage of proliferating cells that released green fluorescence was calculated.

### Flow cytometry assay

The GC cells were seeded into 6-well plates, incubated for 48 h, and then treated with Annexin V-fluorescein isothiocyanate and propidium iodide (Yeasen, Shanghai, China). They were then stained in darkness for 30 min at room temperature. Subsequently, 500 µL of a binding buffer was added to the cells according to protocol. The apoptosis rate was measured using a FACSCalibur flow cytometer.

### Transwell invasion assay

Approximately 1 × 105 GC cells were plated in Matrigel-coated chambers with 8-um pores (BD Biosciences, USA). Subsequently, 600 µL of 20% serum medium was added to the bottom chamber, and the cells in the upper chamber were wiped off. The cells were then fixed using 4% paraformaldehyde and stained using 1% crystal violet. The cells were observed and counted under a microscope.

### Western blot

The protein was extracted using a radioimmunoprecipitation assay (RIPA) Lysis Buffer, separated using sodium dodecyl sulphate-polyacrylamide gel electrophoresis (SDS-PAGE), transferred onto a polyvinylidene difluoride membrane, blocked with 5% skim milk (Beyotime, China) for 1 h, and incubated using the following antibodies at 4 °C overnight: anti-Bcl-2 (1:1000; ab32124, Abcam, USA), anti-BAX (1:5000; cat. no.60267-1, Proteintech, USA), and anti-DHX9 (1:1000; ab183731, Abcam, USA). The membrane was washed, and the secondary antibody was incubated. Finally, the proteins were visualized using ECL (Advansta, USA).

### Fluorescence *in situ* hybridization (FISH) assay

The GC cells were fixed in 4% formaldehyde for 15 min, washed with PBS 2-3 times, treated with pepsin (1% in 10 mmol/L HCl), and dehydrated with ethanol. The samples were then incubated with 40 nmol/L of FISH probe at 80 °C for 2 min, hybridized at 55 °C for 2 h, and dehydrated. Finally, DAPI was added to the air-dried slides for observation. The RNA FISH probes were designed and synthesized by Servicebio (Wuhan, China).

### Luciferase reporter assays

ChIPBase v2.0 (http://rna.sysu.edu.cn/chipbase/index.php) was used to predict the potential TF in the PCAT19 promoter. The SP1-binding motif in the PCAT19 promoter was identified using the JASPAR database (http://jaspar.genereg.net/). Different fragment sequences were designed and inserted into the pGL3-basic vector. Luciferase activity was assessed using a Dual-Luciferase Assay kit (Promega, USA).

### Immunohistochemistry (IHC)

The GC tissue sections were dewaxed with xylene, hydrated with gradient alcohol (100%, 95%, 90%, 75%), and washed with PBS three times. The sections were blocked using 5% goat serum diluted in PBS, and they were incubated using anti-DHX9 antibody at 4 °C overnight. The sections were washed and incubated with horseradish peroxidase-conjugated goat serum, and incubated with secondary anti-rat antibody at 37 °C for 1 h. A DAB mixture was used to stain the sections.

The samples were counterstained, dehydrated, transparentized, and mounted according to the protocol of the manufacturer. Images were captured using a microscope, and the positive expression of DHX9 in the sections showed a yellowish brown color.

### Statistical analysis

Statistical analyses were performed using GraphPad Prism 8.0 and the SPSS 25 software. The associations between PCAT19 and clinical pathologic characteristics were analyzed by Chi-square tests, and the differences between groups were assessed using t-test. The Kaplan-Meier method and log-rank test were used to compare the differences between patient prognoses (P < 0.05).

## Results

### LncRNA PCAT19 expression profile in GC

**Figure 1:**
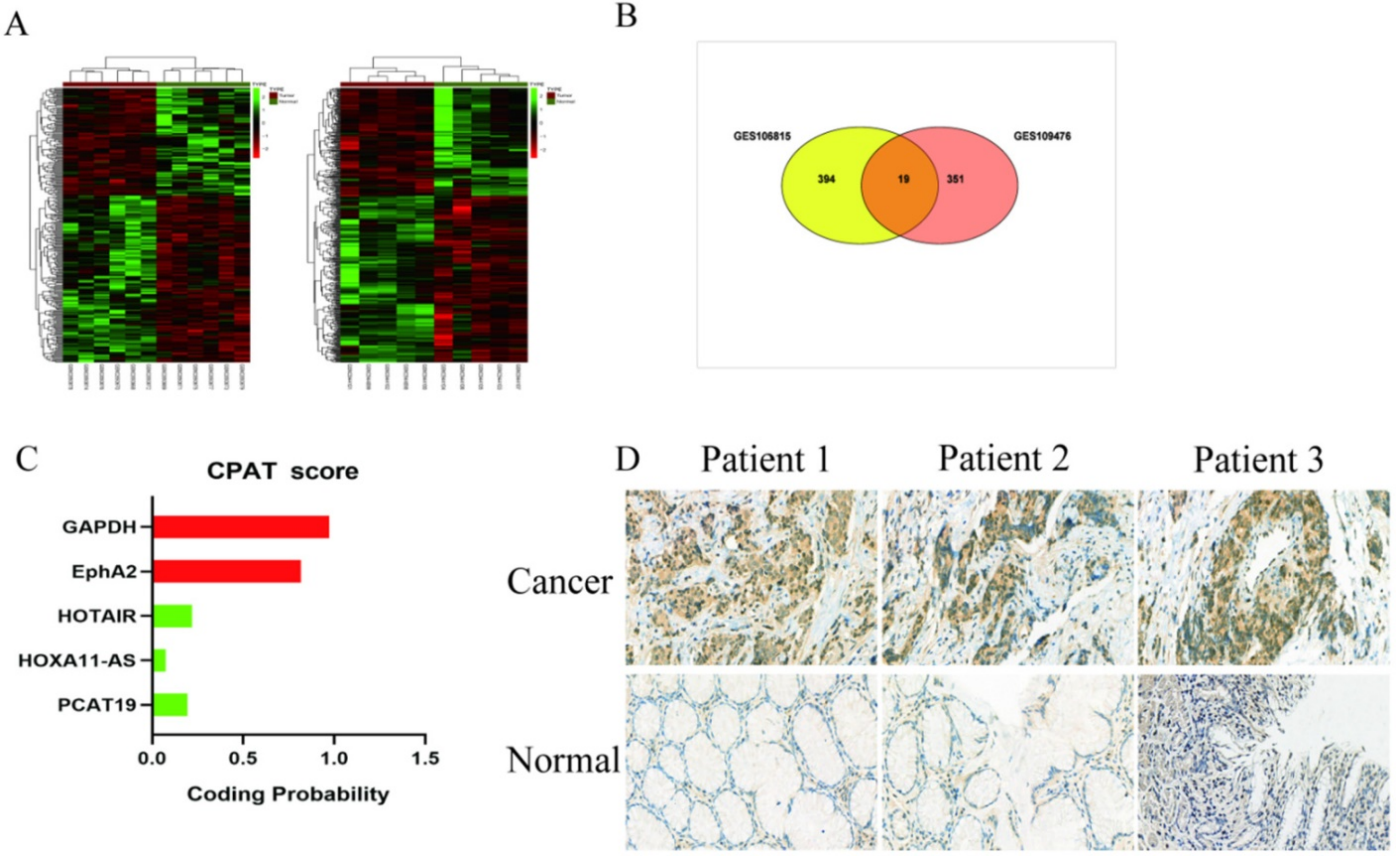
** Expression profile of lnRNA in GC. A.** Hierarchical clustering analysis of lncRNAs that were differentially expressed (fold change > 2; P < 0.05). **B.** Overlap of misregulated lncRNAs in GSE106815 and GSE109476 datasets. **C.** CPAT analyses of the protein-coding potential of PCAT19. **D.** *In situ* hybridization to detect PCAT19 expression in GC tissues and adjacent non-tumor tissues.

We analyzed the microarray datasets GSE106815 and GSE109476 to investigate the differential expression profile of lncRNAs in GC and non-cancer tissues with |logFC| > 2 and P < 0.05. There were 413 lncRNAs misregulated in GSE106815, and 370 lncRNAs misregulated in GSE109476 (**Fig. 1A**). We identified 19 misregulated lncRNAs common to both datasets (**Fig. 1B, Table S2**). Based on the laboratory experimental results, PCAT19 was selected for further investigation. The protein-coding potential of lncRNA PCAT19 was determined using the coding potential assessment tool (CPAT) 12. The CPAT score of PCAT19 was 0.217, which is below 0.5, thereby suggesting that PCAT19 is a non-coding RNA (**Fig. 1C**). To confirm the results of the bioinformatics analysis, the PCAT19 expression was detected by ISH assay, which revealed that PCAT19 was significantly upregulated in GC tissue but not in adjacent normal tissue (**Fig. 1D**).

### Upregulation of lncRNA PCAT19 in GC and correlation with poor prognosis of GC patients

**Figure 2:**
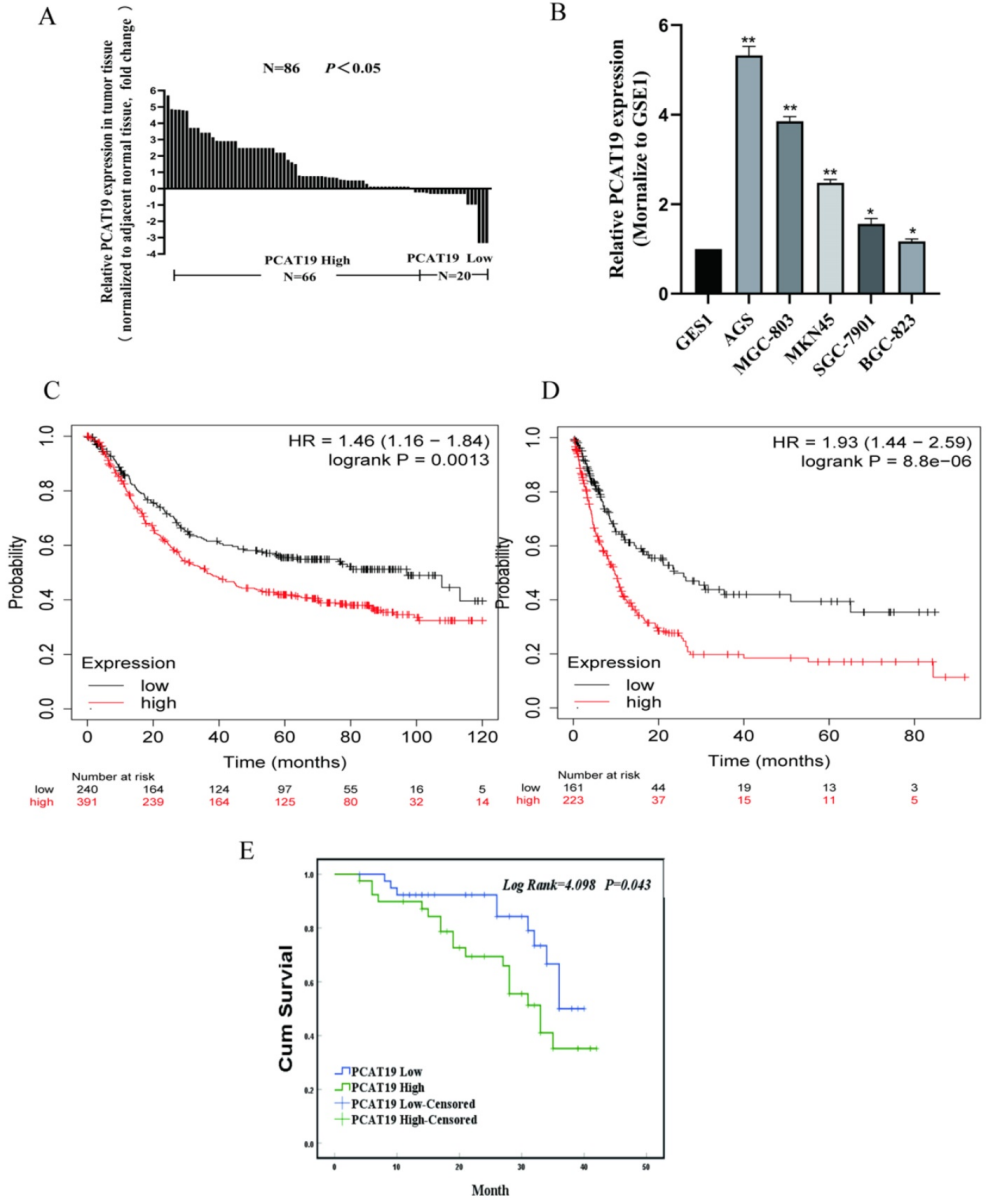
** Overexpression of LncRNA PCAT19 in GC and correlation with poor prognosis of GC patients. A.** Expression of PCAT19 in GC tissues from TCGA database.** B.** Relative PCAT19 expression in GC analyzed by qRT-PCR. **C.** PCAT19 expression levels of GC cell lines and normal GES-1 gastric epithelium cell line by qRT-PCR. **D.** Kaplan-Meier analysis of OS in 86 GC patients. **E and F.** Kaplan-Meier Survival Plots (K-M plots) analysis of OS and disease-free survival (DFS) of GC patients based on PCAT19 expression.

According to The Cancer Genome Atlas (TCGA) database, PCAT19 is significantly upregulated in GC clinical tissues (**Fig. 2A**). To further detect the expression levels of PCAT19 in GC, we conducted qRT-PCR in 86 GC tissues as well as non-cancerous tissues, and the results showed that the PCAT19 expression increased in over 76% of the patients (**Fig. 2B**). Similar results were also observed in the GC cell lines compared to human GES1 gastric mucosal epithelial cells (**Fig. 2C**). We divided the PCAT19 enrichment into different groups according to the median PCAT19 levels of 86 samples to analyze the correlation between PCAT19 expression and patient clinicopathological characteristics. The results showed that higher PCAT19 expression was significantly correlated with larger tumor size (P = 0.041), lymphatic metastasis (P = 0.009), and TNM stage (P = 0.005) (**Table 1**). The Kaplan-Meier analysis also showed that patients in the high PCAT19 group presented a poorer OS rate (median OS: 29.5 months) than those in the low PCAT19 group (median OS: 34.4 months) (**Fig. 2D**). We searched for PCAT19 enrichment in the kmplot databases (**www.kmplot.com**), and the obtained data supported our results 13 (**Fig. 2E and 2F**). However, further analysis of the multivariate Cox proportional hazard model showed that PCAT19 was not an independent prognostic indicator (**Table 2**). Nevertheless, PCAT19 is a potential biomarker of poor prognosis in GC patients.

### PCAT19 potential to promote proliferation and invasion of GC cells

**Figure 3:**
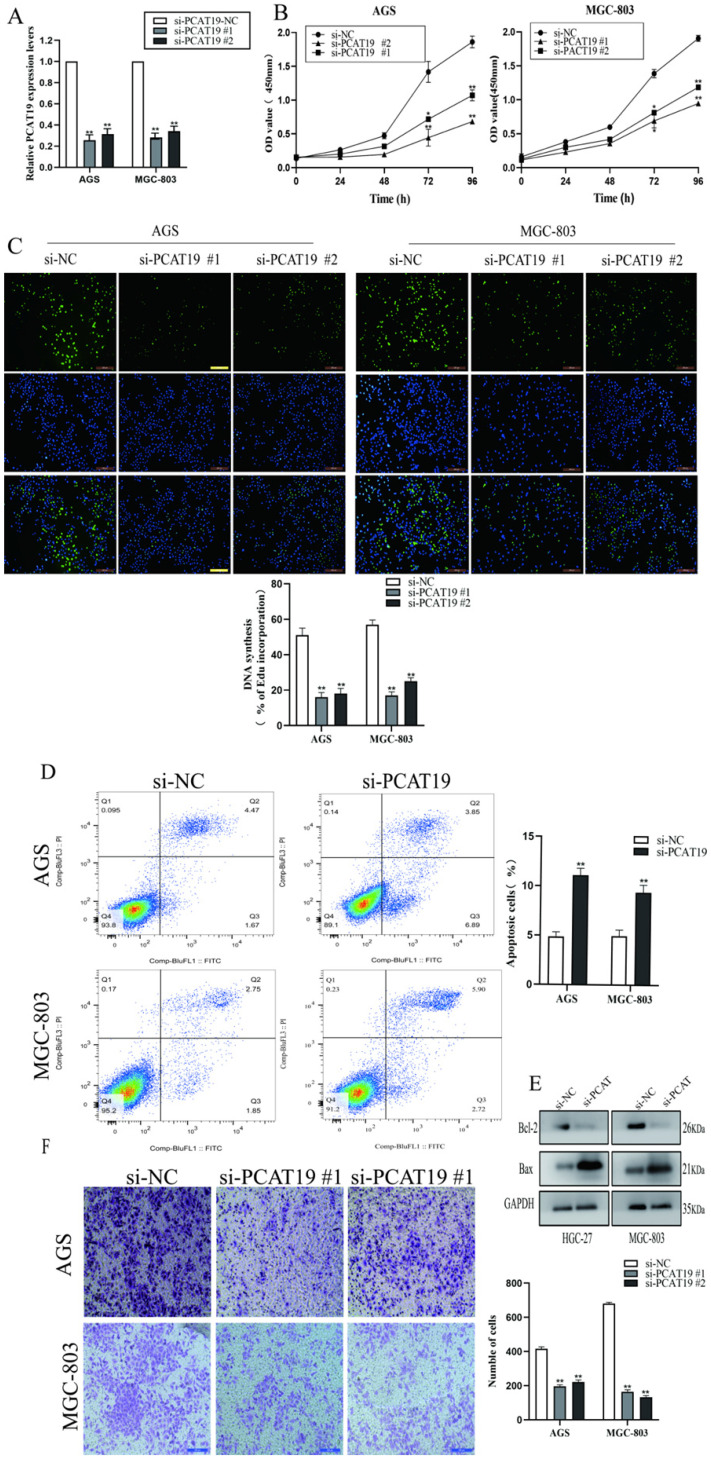
** Proliferation and invasion of GC cells promoted by PCAT19. A.** Transfected efficiency detected by qRT-PCR in AGS and MGC-803. **B.** Use of CCK-8 assays to determine the cell viability of si-PCAT19 and si-NC in GC cells. **C.** Use of EdU assay to assess the proliferation rate of AGS and MGC-803. **D.** FCM of apoptosis in GC cell transfected with si-NC and si-PCAT19. **E.** Levels of Bax and Bcl-2 examined by western blot. **F.** Transwell assay to clarify the invasion of GC cells after transfection of si-PCAT19. *P < 0.05, **P < 0.01.

To further explore the role of lncRNA PCAT19 in GC, we conducted an lncRNA PCAT19 knockdown by siRNA. The transfection efficiency was investigated using qRT-PCR in AGS and MGC-803 cells (**Fig. 3A**). The CCK-8 assay demonstrated that the PCAT19 knockdown impaired cell proliferation (**Fig. 3B**). Similar results were also observed in the EdU assay (**Fig. 3C**). Flow cytometry was performed to detect the effect of PCAT19 on GC apoptosis, and the results revealed that the PCAT19 knockdown significantly increased the apoptosis rate in GC cells (**Fig. 3D**).

Western blotting also demonstrated that PCAT19 significantly reduced the expression of the antiapoptotic protein Bcl-2 and increased the Bax expression (**Fig. 3E**). We also investigated the role of lncRNA PCAT19 in the invasion of GC cells, and the transwell assays showed that it promoted the invasion ability of GC cells (**Fig. 3F**).

### SP1 activated PACT19 transcription in gastric cancer cells

**Figure 4:**
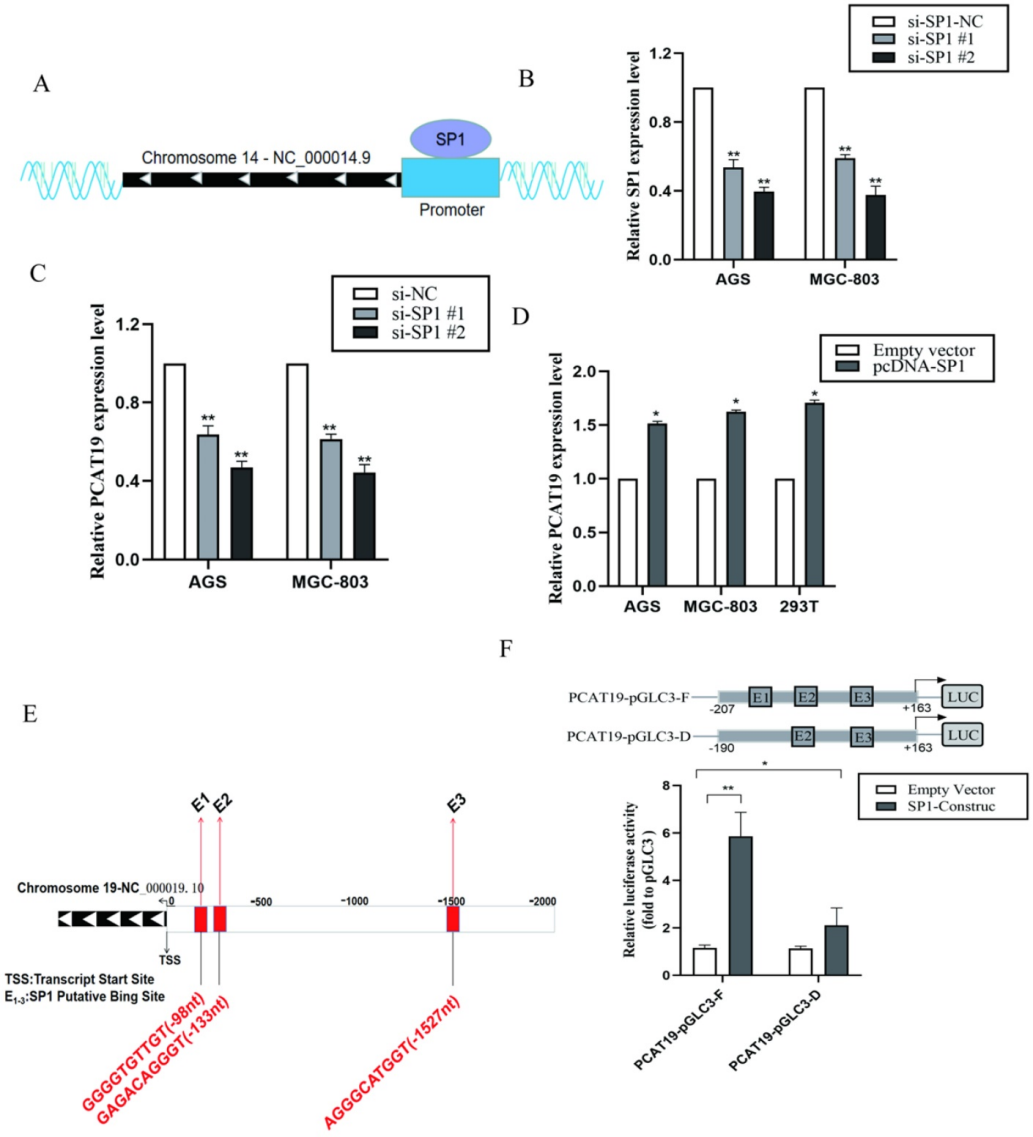
** Promotion of PCAT19 transcription in GC cells by SP1. A.** Schematic outline of the predicted binding of SP1 to PCAT19 gene promoter. **B.** Expression of SP1 detected by qRT-PCR after transfection of siRNA; C: Inhibition of PCAT19 expression by the knockdown of TF SP1 expression in AGS and MGC-803. **D.** PCAT19 expression was detected by qRT-PCR in AGS, MGC-803 and 293T cells transfected with SP1. **E.** Schematic outline of the predicted binding site of SP1 with putative binding sites in the PCAT19 promoter region. **F.** Use of dual-luciferase reporter assays to determine the SP1 binding on the PCAT19 promoter region. *P < 0.05, **P < 0.01.

To determine if PCAT19 regulation can be activated by certain TFs, we searched for potential TF for the PCAT19 promoter in the ChIPBase v2.0 database (**http://rna.sysu.edu.cn/chipbase/index.php**) 14. Approximately 32 TFs were identified as potential TFs of PCAT19. The positive enrichment score was downloaded from the analysis of JASPAR (**http://jaspar.genereg.net; Table S3 and S4**). We observed that TFs SP1 and USF1 were most likely to be involved in regulating the PCAT19 expression because their predicted JASPAR scores were the highest. We also determined a schematic outline of the predicted binding of SP1 to the PCAT19 gene promoter (**Fig. 4A**). However, further investigation indicated that the SP1 knockdown significantly decreased the PCAT19 expression (**Fig. 4B and 4C**), whereas USF1 presented no effect on the PCAT19 expression in GC cells (**Fig. S1A, and S1B**). Upregulation of SP1 also promoted the PCAT19 expression in GC cells (**Fig. 4D**). Moreover, a bioinformatics analysis predicted three SP1 binding sites (PBSs) in the promoter region of PCAT19 (**Fig. 4E**). We constructed a PCAT19 promoter-driven firefly luciferase expression vector, and the results indicated that the vector was significantly induced by SP1 overexpression, and the deletion of the SP1-binding motif E1 significantly impaired the effect of SP1 on PCAT19 transcription activation (**Fig. 4F**). These results indicate that SP1 might bind to this motif to activate the PCAT19 expression.

### PCAT19 bond to miR-429 and regulation of DHX9 expression

**Figure 5:**
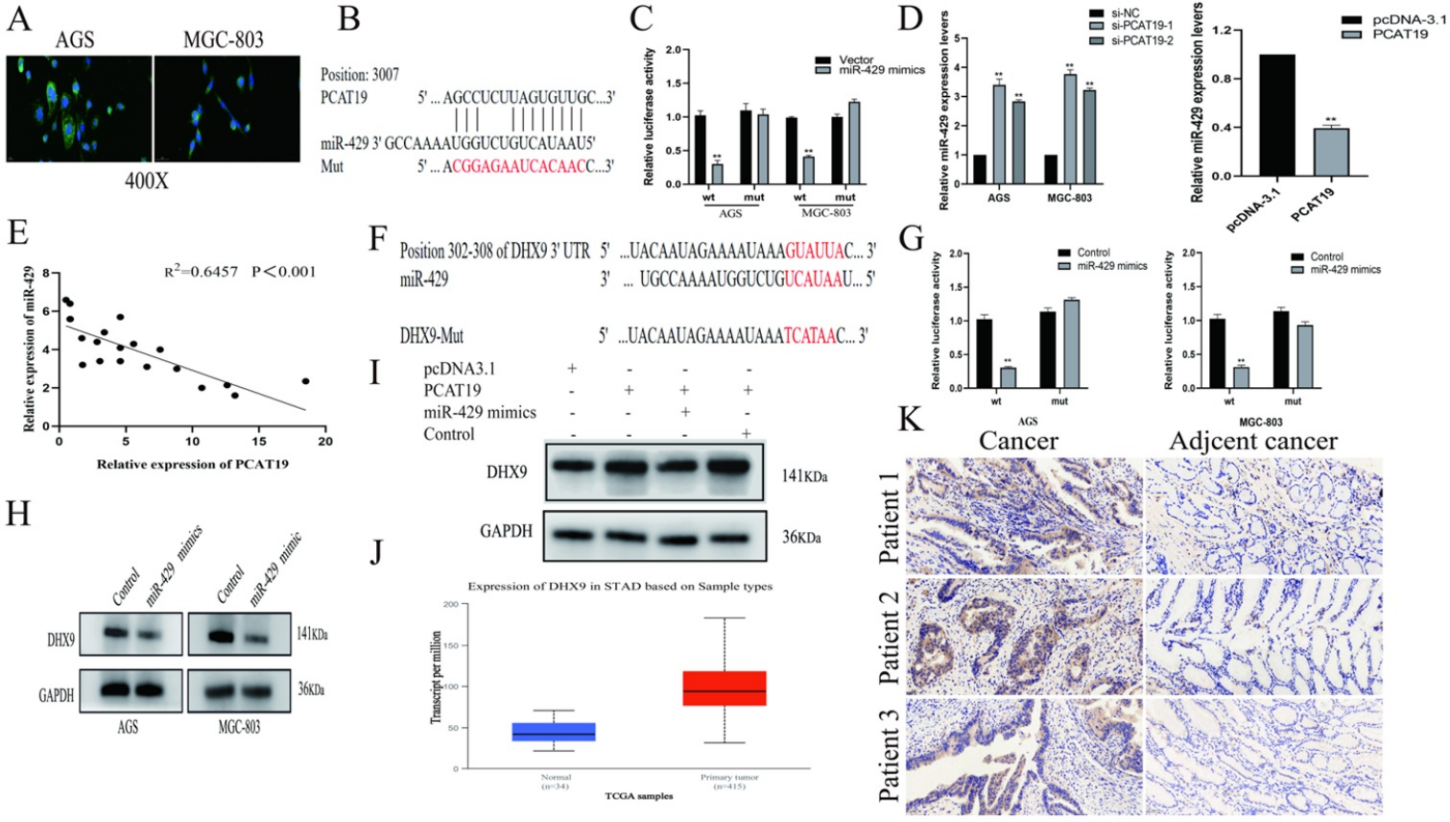
** PCAT19 bond to miR-429 as a sponge and regulation of DHX9 expression. A.** Use of FISH to detect PCAT19 location in AGS and MGC803 cells (green = PCAT19; blue = DAPI). **B.** Potential binding sites between PCAT19 and miR-429. **C.** Dual-luciferase assay to verify the binding of miR-429 to PCAT19. **D.** Expression level of miR-429 detected by real-time PCR after PCAT19 overexpression or knockdown. **E.** Negative correlation between PCAT19 and miR-429 expression in GC tissues.** F.** Prediction of miR-429 binding sites on 3-UTR of DHX9. **G.** Dual-luciferase assay that confirms the binding of miR-429 to DHX9 3-UTR. **H.** Expression level of DHX9 in AGS and MGC-803 cells after transfection of miR-429 mimics.** I.** Expression level of DHX9 after ectopic expression of PCAT19 or/and miR-429. **J.** Expression of DHX9 in GC tissues from the TCGA database. **K.** Expression of DHX9 determined by IHC. *P < 0.05, **P < 0.01.

To further investigate the molecular mechanisms of PCAT19 in GC cells, we examined its distribution in AGS and MGC-803 using a FISH assay. The results revealed that PCAT19 was more prevalent in the cytoplasm (**Fig. 5A**). Several studies have demonstrated that lncRNAs function as ceRNAs for certain miRNAs in the cytoplasm, and the bioinformatics website RNAhybrid-predicted MiR-429 was one of the downstream targets of PCAT19 15 (**Fig. 5B**). To verify our prediction, a dual-luciferase reporter assay was performed, and the results showed that miR-429 reduced the luciferase activity in the PCAT19 site instead of in the PCAT19-Mut. This illustrated the sequence-specific binding of miR-429 to PCAT19 (**Fig. 5C**). In addition, PCAT19 was negatively correlated with miR-429 in qRT-PCR assays (**Fig. 5D**), and a negative correlation was also observed between PCAT19 and miR-429 expression in GC tissues (**Fig. 5E**). We used the TargetScan database (http://www.targetscan.org) to predict the potential target gene of miR-429 and observed that the 3-UTR of DHX9 could bind to it (**Fig. 5F**). Further dual-luciferase reporter assay also demonstrated that miR-429 could specifically reduce the luciferase activity of the UTR of DHX9 (**Fig. 5G**). In addition, the DHX9 expression was negatively downregulated by miR-429 (**Fig. 5H**). To further confirm that PCAT19 regulates the expression of DHX9 by sponging miR-429, we investigated the expression of DHX9 after overexpression of PCAT19. The results confirmed that PCAT19 can upregulate DHX9. However, the expression of DHX9 was reversed after the transfection of miR-429 mimics, but not after the transfection of miR-429 control (**Fig. 5I**). Finally, the TCGA database and IHC staining confirmed that DHX9 was upregulated in clinical GC tissues (**Fig. 5J and 5K**).

## Discussion

Many lncRNAs have been identified via RNA sequencing and have been shown to be involved in the progression of GC 16. Alterations of lncRNAs in GC are already a recognized phenomenon, and it is critical to identify cancer-associated lncRNAs and determine their mechanism of tumor biology 17. However, the molecular mechanism of many lncRNAs related to GC is still poorly understood. In this study, we analyzed the lncRNA profiles of GC using GEO datasets and identified that PCAT19 was related to GC lncRNA. We then examined the PCAT19 expression level using qRT-PCR and ISH assay, and the results showed that PCAT19 increased GC tissue more than adjacent normal tissues. We also analyzed the correlation between PCAT19 and clinicopathologic characteristic of GC patients, and the results confirmed that a higher PCAT19 expression is related to larger tumor size, lymphatic metastasis, and TNM stage. Moreover, patients with higher PCAT19 level presented shorter OS. These results were also confirmed in K-M Plotter analysis including 631 GC patients.

Previous studies have shown that lncRNAs can regulate the malignant phenotypes of cancer cells 18. The PCAT19 knockdown in GC cell lines of AGS and MGC-803 indicated that PCAT19 can inhibit cell proliferation. Zhang et al. 19 presented similar results, which demonstrated that PCAT19 promoted the proliferation of non-small-cell lung carcinoma cells. In addition, PCAT19 inhibited the apoptosis of GC cells. The transwell assay verified the impact of PCAT19 on the invasion of GC cells, and the results suggest that lncRNA PCAT19 promoted the invasion.

As lncRNAs are regulated by TFs such as SP1 and E2F1 20-21, we speculated that there might be a similar modulatory mechanism in the expression of PCAT19. Therefore, we scanned for potential TFs in the PCAT19 promoter and observed that TF SP1 may regulate its expression.

Further examination demonstrated that the SP1 knockdown can decrease the PCAT19 expression level, whereas SP1 overexpression upregulates PCAT19. We then used a dual-luciferase reporter assay to identify special binding motifs of SP1 to the PCAT19 promoter.

Many studies have mechanistically confirmed that lncRNAs function as miRNA sponges in tumorigenesis 22-23. In this study, we examined the PCAT19 distribution in GC cells using FISH assay, and the result showed that PCAT19 was more prevalent in the cytoplasm. In addition, miR-429 was confirmed as a molecular sponge of PCAT19, and it might be regulated by PCAT19. Furthermore, miR-429 also targets DHX9 by miRNA bioinformatics prediction. DHX9 is a member of the DExD/H-box family, and it presents helicases with DEIH sequence at the DExH-box motif 24. In this study, we observed that DHX9 was upregulated in GC tissues, and the TCGA database demonstrated that DHX9 might act as an oncogene in GC. We also confirmed that PCAT19 facilitates the GC development by upregulating DHX9. Therefore, we concluded that PCAT19 functions as miR429 sponge that regulates DHX9 axis.

In summary, our results illustrate that lncRNA PCAT19 is significantly associated with poor prognosis of GC patients. Moreover, this study demonstrates that PCAT19 knockdown can induce apoptosis and inhibit GC cell proliferation, migration, and invasion. Furthermore, PCAT19 expression might be regulated by TF SP1, and it competitively binds to miR-429 and upregulates DHX9 in GC.

## Supplementary Material

Supplementary figure and tables.

## Figures and Tables

**Table 1:** Correlation between PCAT19 expression and clinicopathologic variables of GC patients (N = 86)

Clinicalpathology Characteristic	*n*	PCAT19		χ^2^	*P value*
		Low expression (n=43)	High expression (n=43)		
**Age (years)**					
<60	61	29	32	0.508	0.476
≥60	25	14	11		
**Sex**					
Male	59	32	27	1.350	0.245
Female	27	11	16		
**Tumor size (cm)**					
>5 cm	25	10	15	4.168	0.041*
≤5 cm	61	33	28		
**Histologic differentiation**					
Well and moderate	23	13	10	0.534	0.465
Poor and Undifferentiated	63	30	33		
**Depth of invasion**					
T1+T2	14	10	4	3.071	0.080
T3+T4	72	33	39		
**Lymphatic metastasis**					
Yes	31	19	12	6.880	0.009*
No	55	24	31		
**TNM stage**					
I+II	37	25	12	8.017	0.005*
III+IV	49	18	31		
**Nerve invasion**					
Yes	40	19	21	0.187	0.665
No	46	24	22		
**Vascular invasion**					
Yes	31	14	17	0.454	0.500
No	55	29	26		

**Table 2:** Univariate and multivariate Cox regression analyses of PCAT19 for the DFS of patients (n = 86)

Variables	OS		
	HR	95% CI	P value
Age (< 60 vs. ≥ 60)	0.903	0.411-1.986	0.800
Sex (male vs. female)	0.867	0.595-1.263	0.457
Tumor size (≥ 5 cm vs. < 5 cm)	1.367	0.642-2.908	0.417
Histologic differentiation (well + moderately vs. poorly + undifferentiated)	2.312	0.935-5.720	0.070
Depth of invasion (T3 + T4 vs. T1 + T2)	6.262	0.850-46.162	0.072
Lymphatic metastasis (yes vs. no)	3.890	1.645-9.202	0.002*
TNM stage (III + IV vs. I + II)	3.236	1.987-5.822	0.000*
Nerve invasion (yes vs. no)	1.876	0.901-3.905	0.093
Vascular invasion (yes vs. no)	4.050	1.852-8.854	0.000*
Expression of PCAT19 (high vs. low)	2.150	0.998-4.632	0.050*
**Multivariate analysis**			
Lymphatic metastasis (yes vs. no)	0.849	0.070-10.345	0.898
TNM stage (III + IV vs. I + II)	0.253	0.020-3.234	0.291
Vascular invasion (yes vs. no)	0.174	0.072-0.423	0.000
Expression of PCAT19 (high vs. low)	0.653	0.266-1.603	0.352
