# Supplementary material for: LncRNA PCAT19 induced by SP1 and acted as oncogene in gastric cancer competitively binding to miR429 and upregulating DHX9
Source: J Cancer. 2022 Jan 1;13(1):102–11. doi: 10.7150/jca.61961 (PMC8692695; doi:10.7150/jca.61961)
Supplement: Supplementary file 1 — Supplementary figure and tables. [file jcav13p0102s1.pdf]

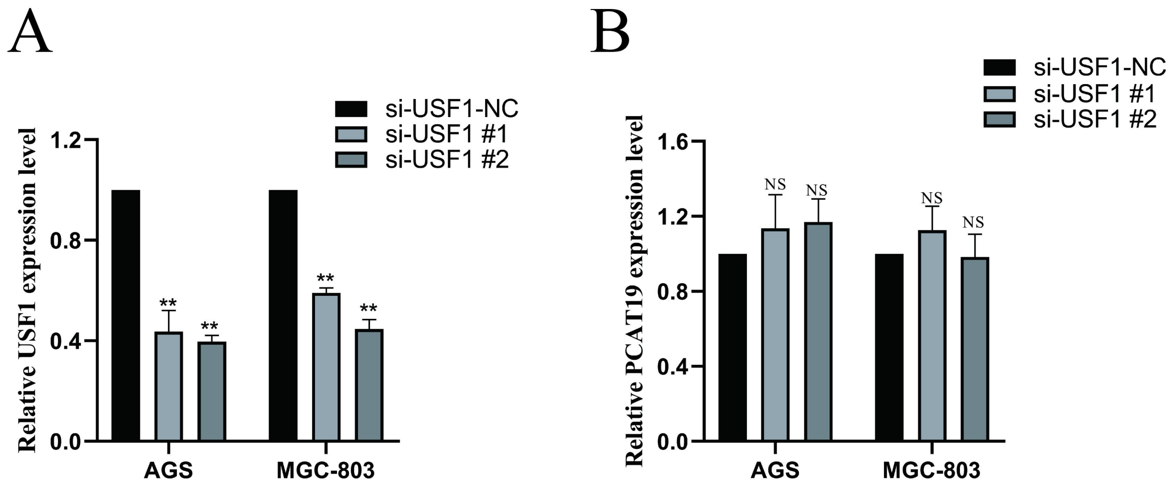

Supplementary Figure 1 USF1 can not activated PACT19 transcription.
A: Expression of USF1 detected by qRT-PCR after transfection of siRNA;
B:Knockdown of TF USF1 expression can not effect the expression of PACT19.

Table S1. Primers used for qRT-PCR,RT-PCR, and siRNAs oligonucleotides

**Primers used for qRT-PCR**	
PCAT19-F	TCAGAACAGGGAACCATTGG
PCAT19-R	CAAGAAGATTCTTATCAGCT
GAPDH-F	GCTCTCTGCTCCTCCTGTTC
GAPDH-R	ACGACCAAATCCGTTGACTC
SP1-F	GCACCTGCCCCTACTGTAAA
SP1-R	GTGCCTCTGTAGCTCATCCG
USF1-F	CTGCTGTTGTTACTACCCAGG
USF1-R	TCTGACTTCGGGGAATAAGGG
miR-429-F	UAAUACUGUCUGGUAAAACCGU
miR-429-R	GGUUUUACCAGACAGUAUUAUU
**siRNAs oligonucleotides**	
si-NC	CUCUCGGAACAUGUCACAU
si-PCAT19 #1	CCUGCAAUUUGGUCUAAAU
si-PCAT19 #1	GCUCUUCUUUAAGCAAUAU
si-SP1-NC	UUCUCCGAACGUGUCACGUATAT
si-SP1 #1	CAGCGUUUCUGCAGCUACCUUGACU
si-SP1 #2	GACAGGUCAGUUGGCAGACUCUACA

Table S2 lncRNAs expression level in GSE106815 and GSE109476 datasets

Ensembl_ID	Symbol	Biotype
ENSG00000238009	AL627309.1	lincRNA
ENSG00000241860	AL627309.5	processed_transcript
ENSG00000228463	AP006222.1	transcribed_processed_pseudogene
ENSG00000237094	AL732372.2	transcribed_unprocessed_pseudogene
ENSG00000230021	AL669831.3	transcribed_processed_pseudogene
ENSG00000231609	AC007098.1	antisense
ENSG00000222041	CYTOR	lincRNA
ENSG00000177738	AC025171.1	sense_overlapping
ENSG00000261568	AL359694.2	lincRNA
ENSG00000253552	HOXA-AS2	antisense
ENSG00000281103	TRG-AS1	antisense
ENSG00000248538	AC022784.1	lincRNA
ENSG00000231052	AL353614.1	lincRNA
ENSG00000224511	LINC00365	lincRNA
ENSG00000262712	AC012676.1	sense_intronic
ENSG00000234899	SOX9-AS1	processed_transcript
**ENSG00000267107**	**PCAT19**	**lincRNA**
ENSG00000237945	LINC00649	antisense
ENSG00000206195	DUXAP8	processed_transcript

Table S3 Potential transcription factor in the PCAT19 promoter

Regulator	Official Full Name	U1kb Sample	D1kb Sample	U1kb Total Site	D1kb Total Site
USF2	upstream transcription factor 1	16	0	16	0
MAX	MYC associated factor X	10	0	10	0
FOS	FBJ murine osteosarcoma viral oncogene homolog	6	0	6	0
FOSL2	FOS-like antigen 2	5	0	5	0
TCF7L2	transcription factor 7-like 2 (T-cell specific, HMG-box)	5	0	5	0
ESR1	estrogen receptor 1	4	0	4	0
FOXM1	forkhead box M1	4	0	4	0
JUND	jun D proto-oncogene	4	0	4	0
E2F6	E2F transcription factor 6	3	0	3	0
NFIC	nuclear factor I/C (CCAAT-binding transcription factor)	3	0	3	0
NKX2-1	NK2 homeobox 1	3	0	3	0
USF2	upstream transcription factor 2, c-fos interacting	3	0	3	0
ESR2	estrogen receptor 2 (ER beta)	2	0	2	0
FOSL1	FOS-like antigen 1	2	0	2	0
JUN	jun proto-oncogene	2	0	2	0
MYC	v-myc avian myelocytomatosis viral oncogene homolog	2	0	2	0
NFYB	nuclear transcription factor Y subunit beta	2	0	2	0
SP1	Sp1 transcription factor	2	0	2	0
ZNF384	zinc finger protein 384	2	0	2	0
ATF3	activating transcription factor 3	1	0	1	0
E2F2	E2F transcription factor 2	1	0	1	0
E2F4	E2F transcription factor 4, p107/p130-binding	1	0	1	0
FOXA1	forkhead box A1	1	0	1	0
FOXA2	forkhead box A2	1	0	1	0
GABPA	GA binding protein transcription factor alpha subunit	1	0	1	0
KDM5A	lysine (K)-specific demethylase 5A	1	0	1	0
MECOM	MDS1 and EVI1 complex locus	1	0	1	0
NR2F2	nuclear receptor subfamily 2 group F member 2	1	0	1	0
PBX1	pre-B-cell leukemia homeobox 1	1	0	1	0
SOX2	SRY-box 2	1	0	1	0
TCF12	transcription factor 12	1	0	1	0

Table S4 Positive enrichment score of TFs in PCAT19 from JASPAR analysis

Name	Score	Relative score	Sequence ID	Start	End	Strand	Predicted sequence
**SP1**	**8.5066**	**0.88358664**	**NC_000019.10:c41454168-41452169**	**1527**	**1536**	**+**	**AGGGCATGGT**
**SP1**	**7.71783**	**0.856073107**	**NC_000019.10:c41454168-41452169**	**98**	**107**	**+**	**GGGGTGTTGT**
SP1	7.19766	0.837928744	NC_000019.10:c41454168-41452169	95	104	+	TTGGGGGTGT
**SP1**	**6.62561**	**0.817974759**	**NC_000019.10:c41454168-41452169**	**133**	**142**	**+**	**GAGACAGGGT**
SP1	6.33692	0.807904598	NC_000019.10:c41454168-41452169	1288	1297	+	AGGGCAGGGC
SP1	6.33692	0.807904598	NC_000019.10:c41454168-41452169	1522	1531	+	AGGGCAGGGC
SP1	6.15904	0.801699912	NC_000019.10:c41454168-41452169	1185	1194	+	GAGGGCTGGT
USF1	7.79957	0.872967864	NC_000019.10:c41454168-41452169	996	1002	+	CATGTGA
USF1	7.0683	0.847803352	NC_000019.10:c41454168-41452169	426	432	+	CAGGTGG
USF1	7.0683	0.847803352	NC_000019.10:c41454168-41452169	1403	1409	+	CAAGTGG
USF1	7.02	0.846141084	NC_000019.10:c41454168-41452169	417	423	+	CACCTGG
USF1	6.50057	0.82826659	NC_000019.10:c41454168-41452169	739	745	+	CAAGTGA
USF1	6.50057	0.82826659	NC_000019.10:c41454168-41452169	1237	1243	+	CAAGTGA
USF1	5.79746	0.804071201	NC_000019.10:c41454168-41452169	914	920	+	CAGGTGT
USF1	5.79746	0.804071201	NC_000019.10:c41454168-41452169	1482	1488	+	CAAGTGT
E2F1	6.00106	0.858305906	NC_000019.10:c41454168-41452169	1513	1523	+	GGAGCAGGAAG
E2F1	5.40621	0.849356656	NC_000019.10:c41454168-41452169	1591	1601	+	AACCCGGGAGG
E2F1	5.1546	0.845571261	NC_000019.10:c41454168-41452169	1119	1129	+	ACCGCGCCAGG
E2F1	4.47704	0.835377435	NC_000019.10:c41454168-41452169	1377	1387	+	GAGGAGGCAGC
E2F1	4.47704	0.835377435	NC_000019.10:c41454168-41452169	1380	1390	+	GAGGCAGCAGC
MAX	4.18849	0.834033592	NC_000019.10:c41454168-41452169	414	423	+	AGTCACCTGG
MAX::MYC	8.76116	0.820022275	NC_000019.10:c41454168-41452169	423	433	+	GTCCAGGTGGT
E2F4	5.78509	0.83642348	NC_000019.10:c41454168-41452169	1514	1524	+	GAGCAGGAAGG
E2F4	5.12795	0.826016976	NC_000019.10:c41454168-41452169	1592	1602	+	ACCCGGGAGGT
E2F4	4.30553	0.812993124	NC_000019.10:c41454168-41452169	684	694	+	AGGCTGGAATG
E2F4	4.09461	0.80965305	NC_000019.10:c41454168-41452169	1120	1130	+	CCGCGCCAGGC
E2F4	3.81028	0.805150294	NC_000019.10:c41454168-41452169	1936	1946	+	TGGCTGGAGTG
TCF7L2	10.1342	0.882493524	NC_000019.10:c41454168-41452169	1263	1276	+	AAAGCTCAAAGCAG
TCF7L2	8.25335	0.856499603	NC_000019.10:c41454168-41452169	1311	1324	+	TGTGATGAAAGTAT
TCF7L2	6.36538	0.830407293	NC_000019.10:c41454168-41452169	1738	1751	+	AATAATCAAAACCA
TCF7L2	6.04023	0.825913671	NC_000019.10:c41454168-41452169	1694	1707	+	AAAGAAAAAAAGAA
TCF7L2	5.99609	0.825303571	NC_000019.10:c41454168-41452169	1673	1686	+	AAACAAAAAAAGAA
TCF7L2	5.76571	0.822119667	NC_000019.10:c41454168-41452169	557	570	+	AGACTTAAGAGGGT
TCF7L2	5.11784	0.813165886	NC_000019.10:c41454168-41452169	1256	1269	+	ATAGGTCAAAGCTC
TCF7L2	4.37416	0.802888029	NC_000019.10:c41454168-41452169	1146	1159	+	ACAGAAGAAAATAA
TCF7L2	4.27682	0.801542837	NC_000019.10:c41454168-41452169	1088	1101	+	CCTCATCACAGAGG
TP63	13.0014	0.878762743	NC_000019.10:c41454168-41452169	782	801	+	AGGCATGCACCACCACGCCC
TP63	8.92861	0.832031784	NC_000019.10:c41454168-41452169	54	73	+	AAATTTGCATAGGCCTGCTC

TP63	7.95678	0.820881032	NC_000019.10:c41454168-41452169	266	285	+	CACCATGCCCAGCCCTGGAC
